# Prospective comparison of the anterior and lateral approach in hemiarthroplasty for hip fractures: a study protocol

**DOI:** 10.1186/s12891-017-1724-9

**Published:** 2017-08-23

**Authors:** Max P. L. van der Sijp, Inger B. Schipper, Stefan B. Keizer, Pieta Krijnen, Arthur H.P. Niggebrugge

**Affiliations:** 1 Department of Surgery, Haaglanden Medical Centre, P.O. Box 432, 2501 CK The Hague, the Netherlands; 2 Department of Surgery, Leiden University Medical Centre, P.O. Box 9600, 2300 RC, Leiden, the Netherlands; 3 Department of Orthopaedics, Haaglanden Medical Centre, P.O. Box 432, 2501 CK The Hague, the Netherlands

**Keywords:** Proximal femoral fracture, Anterior approach, Lateral approach, Hemiarthroplasty, Hip prosthesis, Rehabilitation, Functionality

## Abstract

**Background:**

The Direct Anterior Approach (DAA) is an alternative approach to the currently most used Lateral Approach (LA) for hip replacement in femoral neck fracture patients. Compared to the LA, the DAA minimizes soft tissue damage. Sparing muscle tissue may facilitate early and improved postoperative mobility. It may also be associated with fewer complications, increased quality of life and lower 1-year mortality. The aim of this study is to compare postoperative complications, hip function and patient mobility after hemiarthroplasty via the anterior or lateral approach following a displaced femoral neck fracture.

**Methods:**

138 elderly patients with displaced femoral neck fractures will be operated using either the direct anterior approach or the lateral approach for a hemiarthroplasty in a single centre, prospective, comparative cohort study. The choice of surgical approach will depend on the expertise of the trauma surgeon on call. The primary outcome of this study will be the functionality of the hip after surgery measured using the Harris Hip Score during routine outpatient check-ups. Secondary outcomes include surgical and non-surgical complication rates, admission time, postoperative pain, rehabilitation time, performance in activities of daily living, health-related quality of life measured, cognitive function and balance.

**Discussion:**

Many approaches are known for hip replacement arthroplasty in trauma patients with little consensus on the preferred method. Identifying the best approach facilitating an adequate and fast recovery could optimize patient independence and quality of life and minimize rehabilitation costs, morbidity and mortality rates. The study design will reflect daily clinical practice and aims to present an accurate depiction of clinical outcomes.

**Trial registration:**

This trial entered the Dutch Trial Registry with registration number (NTR)6238 on the 24th of April 2017. http://www.trialregister.nl/trialreg/index.asp. Protocol version 2.0 16–03-2017.

## Background

Femoral neck fractures are amongst the most common fractures in the elderly population [1]. Treatment requires immediate hospitalization, surgical treatment and intensive physical therapy aimed at achieving prefracture levels of function and mobility [2]. Although hip arthroplasty enables early postoperative mobilization, rehabilitation is still required due to the invasive nature of the procedure and the frailty of the average patient [3]. Fast recovery is desirable as prolonged rehabilitation is associated with high morbidity and mortality rates and increased healthcare costs [4, 5].

Currently one of the most commonly used approaches for hip replacement arthroplasty is the Lateral Approach (LA), also known as the Straight Lateral, the Direct Lateral, the Hardinge or the Transgluteal Approach, which provides excellent exposure of both the proximal femur and acetabulum, but requires partial dissection of the gluteus medius muscle insertion for an adequate exposure of the capsule [6]. Consequently it is associated with postoperative abductor muscle dysfunction. This may cause a limp or Trendelenburg gait in patients with reduced abductor strength, as well as greater trochanteric pain or tenderness related to muscle injury, and dislocations of the hip [6, 7]. These adverse outcomes may be prevented by using an alternative approach, the Direct Anterior Approach (DAA), also known as the Anterior Minimal Invasive Surgery (AMIS), the Anterior Supine Intermuscular (ASI), the (modified) Smith-Petersen or the Hueter approach. The DAA is used by an increasing number of orthopaedic surgeons for (non-traumatic) Total Hip Arthroplasty (THA). Recent studies indicate that the muscle-sparing nature of this technique is beneficial to the patient’s recovery and reduces the incidence of complications [6]. Although many subtle variations of the DAA have been described, the common goal is to leave muscle tissue intact and approach the hip joint through the intermuscular spaces of the tensor fasciae latae, the sartorius, the rectus femoralis and the gluteus medius [8]. Disadvantages of the DAA include limited exposure of the hip joint which can cause difficulties in correct alignment of the femoral arthroplasty, especially in obese patients, and perioperative complications in osteoporotic bone [6]. Valgus position of the implant is a well-known pitfall of this procedure [3], as is damaging the lateral femoral cutaneous nerve [9].

Studies comparing approaches of the hip joint for THA often favour the DAA for its low dislocation rates and intact abductor function [6, 10]. Despite differences in the procedures and patient populations of THA and hemiarthroplasty (HA), a faster and better recovery with fewer complications is expected for both procedures when using a less invasive approach [10]. A few studies concerning the DAA for HA suggest benefits of the DAA over the LA, but large comparative prospective trials are lacking [11].

Sparing muscle tissue and using a less invasive approach could lead to improved postoperative mobility, less permanent institutionalization, increased quality of life, fewer complications and lower long term mortality. If clinical benefits of the DAA approach outweigh its disadvantages, this approach could be preferable over the LA for HA after displaced hip fractures.

### Objective

The aim of this study is to compare hip function, postoperative complications and patient mobility after hemiarthroplasty via the anterior or lateral approach for a displaced femoral neck fracture in elderly patients.

## Method/design

This single centre prospective comparative cohort study is part of the ‘Hip Fracture Centre’ (HFC) project that prospectively documents the treatment outcomes of all consecutive hip fracture patients admitted to the Haaglanden Medical Centre (HMC) Bronovo in The Hague, the Netherlands. The HMC annually treats more than 500 patients with proximal femoral fractures and is part of a major trauma centre collaboration in the western part of the Netherlands. The HFC project is a multidisciplinary project designed to improve the care for hip fracture patients in our hospital.

### Study population

All consecutive patients 70 years or older admitted to the study hospital with an X-ray proven displaced femoral neck fracture (AO type 31 B1-B3), treated with a cemented hemiarthroplasty who are considered able to rehabilitate will be included in this study. Eligibility for rehabilitation is determined during hospitalization for all patients in twice weekly assessments by a multidisciplinary team, including a trauma surgeon, ward doctor, trauma nurse, physiotherapist, dietician, geriatrician and a transfer nurse. Patients are considered fit for rehabilitation when they can participate in physiotherapy by being physically and mentally able to adequately follow instructions. Patients fit for rehabilitation are either discharged home with ambulatory physiotherapy, or to a geriatric rehabilitation care institute. Patients with an active lifestyle and unrestricted mobilization without walking aids are considered eligible for total hip arthroplasty by the treating trauma surgeon and are excluded from the study. Inclusion and exclusion criteria are presented in Table 1.

**Table 1:** Inclusion and exclusion criteria for eligible study subjects

Inclusion criteria	Exclusion criteria
Age ≥ 70 years	Eligible for total hip arthroplasty^a^
X-ray proven displaced femoral neck	Treated with an uncemented hemiarthroplasty
Fracture (AO type 31 B1-B3)	Incapable of rehabilitation due to severe cognitive impairment
Able to rehabilitate	Incapable of rehabilitation due to pre-existing physical restrictions.
	Concomitant traumatic comorbidities restricting long-term rehabilitation.

^a^Patients with an active lifestyle and unrestricted mobilization without walking aids

### Outcomes

#### Primary outcome

The primary outcome of this study is the assessment of daily life functionality of the hip fracture patient after cemented hemiarthroplasty using the Harris Hip Score (HHS).

#### Secondary outcomes

##### Clinical outcomes

- Surgical parameters: mean operation time (skin-to-skin), mean total blood loss.
- Surgical complication rates (postoperative bleeding,^1^ haematoma formation, implant failure, implant dislocation,^2^ implant luxation, femoral head necrosis, periprosthetic fractures,^3^ superficial wound infection,^4^ deep wound (prosthesis) infection,^5^ nerve damage^6^).
- Cognitive status measured with the 6CIT score [12, 13] at admission to the hospital and at 6 weeks, 3 months and 12 months after surgery, and DOS scores during admission [14].
- Duration of hospital stay, cause of delayed discharge (considered later than 72 h after surgery), discharge destination and duration of rehabilitation (in days).
- Readmission and operative revision rate.
- 1-year mortality.^7^
- Non-surgical complications up to 12 months after surgery (delirium,^8^ anaemia,^9^ cardiac complications (decompensation and ischemia), CVA, pressure sores,^10^ electrolyte disturbances, pulmonary embolism,^11^ pneumonia,^12^ renal failure, sepsis,^13^ deep venous thrombosis^14^ and urinary tract infections^15^).
- Functionality and balance through a series of physiotherapeutic tests (Short Physical Performance Battery [15], Timed Up and Go test [16], Functional Ambulatory Categories [17]) at 6 weeks, 3 months and 12 months after surgery.
- Pain measured using a visual analogue scale (VAS) during in-hospital treatment and at 6 weeks, 3 months and 12 months after surgery.
- Patient-reported performance in activities of daily living using the Katz ADL Index [18] at 6 weeks, 3 months and 12 months after surgery.
- Health-related quality of life using the EQ-5D questionnaire [19, 20] at 6 weeks, 3 months and 12 months after surgery.

### Baseline parameters

Additional parameters include: age, sex, date of birth, general health score (using the ASA classification [21]), fracture type and side, date and time of admission and surgery, surgeon’s experience level, type of anaesthesia, surgical approach, Body Mass Index, preoperative cognition state (using the 6CIT score), preoperative nutritional state (using the SNAQ [22]) and use of osteoporosis medication. The prefracture performance in activities of daily living (using the Katz ADL) and health-related quality of life (using the EQ-5D) before the fracture will be assessed during admission as a baseline measure.

### Treatment and procedures

Patients admitted to the hospital with a proximal femoral fracture will be treated according to the treatment guidelines for proximal femoral fracture in the elderly of the Nederlandse Vereniging voor Heelkunde (Dutch trauma surgery society) [23]. A timeline with all scheduled data registrations and assessments is presented in Table 2 *.*

**Table 2:** Timeline for scheduled procedures and assessments

Procedure/Assessment	Admission	In-hospital treatment phase	6 weeks after surgery^a^	3 months after surgery^a^	12 months after surgery^a^
*Baseline measurements*	X				
*Physical examination*	X	X	X	X	X
*X-ray assessment*	X				
*Katz ADL*	X		X	X	X
*EQ-5D*	X		X	X	X
*6CIT*	X		X	X	X
*SNAQ*	X				
*VAS pain*		X	X	X	X
*Complication* ^*a*^ *registration*		X	X	X	X
*Mobility tests* ^*b*^			X	X	X

^a^Any patient reported complication in the previously described surgical and non-surgical complication list and any reason for a postoperative readmission to a hospital

^b^HHS, SPPB, TUG, FAC

#### Preoperative procedures

In accordance with standard care, X-ray examinations of the pelvis, hip and thorax are made on admission and assessed by the radiologist and orthopaedic trauma surgeon. After diagnosis all patients will receive an information brochure about the standard care (treatment and rehabilitation) of the study hospital. All patients are screened for osteoporosis and treated if necessary.

At this stage, registration of patient data will be performed by the physician on call in the emergency department (ED) and the ED nurse. The baseline questionnaires (including the SNAQ and Katz ADL and the EQ-D5) are registered by a proficient nurse upon arrival on the surgical ward before surgery.

#### Surgical procedures

Surgery will be performed by experienced surgeons in accordance with the national surgical treatment protocol for proximal femoral fracture of the Nederlandse Vereniging voor Heelkunde (Dutch trauma surgery society). The choice of a specific surgical approach will depend on the experience of the surgeon on call: all surgeons use the approach they are most familiar with. The group consists of 16 highly experienced orthopaedic trauma surgeons. Residents with insufficient experience to perform the operation independently, operate under strict supervision of the trauma surgeons. The LA and DAA are standard approaches for hemiarthroplasty in the study hospital.

Patients are operated as soon as possible after full medical check-up (including laboratory tests, physical examination and consultation by a geriatric specialist) and anaesthesiological approval.

The prosthesis used will be the self-centring, 3-point-contact anchorage cemented CCA straight stem prosthesis in accordance with Prof. Müller in a standard of lateral version combined with a short or medium hemiprosthesis head produced by of Mathys Medical®. Patients with a severe history of cardiovascular diseases with pump failure or hypotension during operation are at risk of adverse events associated with bone cement implantation syndrome and are excluded from the study and treated with an uncemented prosthesis. This is assessed and decided subjectively by the treating anaesthesiologist and trauma surgeon.

The anterior approach will be performed with the patient in a supine position. An incision of about 15 cm is made in a straight line starting two fingers lateral and caudal of the spina iliaca anterior superior heading towards the lateral side of the patella. The fascia is dissected between the m. tensor fascia lata and the m. sartorius. Using blunt dissection of the intermuscular space, the crossing vessels are cauterized and dissected. The capsule is excised to expose the joint. After completion of the procedure, the wound is closed in layers.

The lateral approach is performed with the patient in a lateral position. An incision is made starting 8 cm distal of the tip of the greater trochanter upwards to the tip and 5 cm proximally of the tip in a slightly dorsal direction, exposing the fascia lata. The fascia lata is split and retracted to expose the insertion of the m. gluteus medius which is dissected with or without vastogluteal continuity. A T-shaped incision is made in the capsule to expose the hip joint. After completion of the procedure, the capsule is closed and the wound is closed in layers.

#### Postoperative procedures

Postoperative patients are visited daily by a surgical resident and discussed twice a week by a multidisciplinary team including an orthopaedic trauma surgeon, ward doctor, trauma nurse, physiotherapist, dietician, geriatrician and transfer nurse. In our study hospital, hip fracture patients are preferably discharged 3 days after surgery according to local protocol. Patients are discharged home if pain is manageable, no active complications are present, and if mobility is adequate for living independently (meaning that the patient can safely make indoor transfers) or if home care is available and sufficient. If home care is not sufficient, discharge to one of the nursing homes specializing in hip fracture rehabilitation is planned. These assessments are also made by the multidisciplinary team for all patients twice a week. All patients fit for rehabilitation will be invited for outpatient check-ups 6 weeks, 3 months and 12 months after surgery. The reasons for failure to follow up will be recorded.

The outpatient check-ups include consultations by a trauma surgeon or surgical resident, a physiotherapist for mobility assessments and a geriatrician for strength and balance assessments and osteoporosis screening and treatment. A timeline with the scheduled procedures and assessments is presented in Table 2.

### Sample size

Reported one-year postoperative results for the HHS in hemiarthroplasty patients vary between 68 (standard deviation [SD] 20 [24] and 78 (SD 10) [25]. Long-term (12 months) postoperative HHS results for specific surgical approaches are lacking in literature. For the sample size calculations a difference in functionality of 10 points as measured by the HHS was considered clinically relevant [26], with a standard deviation of 15. To detect this difference two equally sized cohorts of at least 41 patients with a complete 12 months follow-up are necessary (α = 0.05, power = 80%). Anticipating failure to follow up of 40%, inclusion of at least 69 patients per intervention group, or 138 in total, is required.

### Statistical analysis

Demographic and clinical characteristics of the cohorts will be described using summary statistics and will be compared by univariate analysis. Categorical variables will be compared using the chi-squared test if the data are sufficiently large (expected cell counts >5) or the Fisher’s exact test if this requirement cannot be met. Continuous variables will be compared by unpaired t-tests for normally distributed data, and by the Wilcoxon rank sum test for not normally distributed data.

Outcome parameters will be compared univariably between cohorts, but correction for confounding factors is of major importance because the patients in this study are not randomized. Since the number of study participants is limited relative to the number of potential confounders, a propensity score will be calculated from the available baseline parameters. The propensity score will be used to adjust for differences between the two cohorts in multivariable linear regression analyses for continuous outcome parameters and in multivariable logistic regression analyses for binary outcomes. Repeated outcome measures such as the HHS and pain score will also be analysed using Generalized Linear Models. *P*-values <0.05 will be considered statistically significant. All statistical analyses will be performed using IBM SPSS Statistics version 20.0 or higher.

### Ethical considerations and safety

Patient data will be handled according to the Good Research Practice guidelines. Data will be registered directly and simultaneously with the digital patient files and during oral questionnaires. Data will be registered anonymously in the database to ensure confidentiality. The identification code list will be safeguarded by the principal investigator.

All patients will be treated according to regular protocol in the study hospital. Participation in this observational study will not pose any additional risks for patients and does not influence their treatment in any way. All patients will be given the explicit opportunity to withdraw their data from the study. No individual participant’s data will be published and no informed consent for participation will be collected from the patients. This study was approved by the institutional Medical Research Ethics Committee (METC Southwest Holland; protocol number 16–059). The results of the study will be published in a peer-reviewed medical journal.

## Discussion

Many approaches are known for hip replacement arthroplasty in trauma patients. There is little consensus on the preferred method, which varies per hospital, per surgical team and even per surgeon. Identifying the best possible approach for hemiarthroplasty surgery could lead to shorter admission times, faster rehabilitation and better functional outcomes for elderly patients. Consequently, patient independence could improve, and healthcare costs could be reduced.

Our non-randomized study design implies that each surgical team will use the approach they are most familiar with. The outcome will therefore reflect everyday clinical practice.

### Study status

The prospective collection of data of all consecutive hip fracture patients in the ‘Hip Fracture Centre’ (HFC) care reform project at the Haaglanden Medical Centre Bronovo in The Hague, the Netherlands, was started 1 January 2017. The data collection of the required patient sample is expected to be completed in 2018.

## Abbreviations

6CIT: Six item cognitive impairment test
DAA: Direct Anterior Approach
DHFA: Dutch hip fracture audit
DOS: Delirium observational screening (score)
ED: Emergency department
EQ-5D: EuroQol-5D questionnaire on health status
FAC: Functional ambulation categories
HA: Hemiarthroplasty
HHS: Harris hip score
Katz ADL: Questionnaire on functionality in activities of daily living
LA: Lateral approach
SNAQ: Simplified nutritional appetite questionnaire
SPPB: Short physical performance battery
THA: Total hip arthroplasty
TUG: Timed Up and Go (test)
VAS: Visual analogue scale (for pain)

## Footnotes

1 Postoperative bleeding is defined as any blood loss from the operated area.

2 Implant dislocation is defined as the dislocation of the femoral head from the acetabulum.

3 Periprosthetic fractures are defined as any visual or X-ray confirmed fractures formed during or after surgery with a fracture-plane in direct contact with the prosthesis.

4 Wound infection is defined as the presence of the collective calor, dolor, rubor, tumor and pus formation observed by the treating physician. No positive culture is required.

5 Fistula or pus formation surrounding the prosthesis *and/or*: two or more positive preoperative cultures of identical micro-organisms *or* one preoperative puncture culture with one peroperative culture with an identical micro-organism *or* one positive preoperative or peroperative culture with a virulent micro-organism (*S. aureus*) *or* a suspected clinical assessment.

6 Nerve damage includes any loss of sensory or motor functions associated with the operation area.

7 Mortality is defined as the death of a patient due to any cause.

8 Delirium is defined as a change in consciousness and diagnosed using the DSM-IV criteria.

9 Anaemia is defined as a blood haemoglobin level < 6.0.

10 Pressure sores are sores developed postoperatively on specific locations due to a prolonged bedridden condition of the patient.

11 Pulmonary embolism diagnosis requires CT scan confirmation.

12 Pneumonia is defined as any respiratory symptoms and confirmed by pulmonary infiltration evident in radiological diagnostics.

13 Sepsis is defined as postoperative SIRS (≥2 of the following: (1) a body temperature of ≥38 °C or ≤36 °C, (2) a heart-rate of ≥90/min, (3) a respiratory rate of ≥20/min or (4) a white blood cell count of ≥12.000/mm^3^ or 4.000/mm^3^ or 10% immature neutrophils).

14 Deep venous thrombosis diagnosis requires positive ultrasound or phlebography.

15 Urinary tract infections are defined as infections of the urinary tract confirmed by nitrite test, dipslide or urine culture.
